# 3,4-Dichlorophenoxyacetate interleaved into anionic clay for controlled release formulation of a new environmentally friendly agrochemical

**DOI:** 10.1186/1556-276X-8-362

**Published:** 2013-08-23

**Authors:** Sheikh Ahmad Izaddin Sheikh Mohd Ghazali, Mohd Zobir Hussein, Siti Halimah Sarijo

**Affiliations:** 1 Faculty of Applied Science, Universiti Teknologi MARA, Shah Alam, Selangor 40450, Malaysia; 2 Material Synthesis and Characterization Laboratory (MSCL), Institute of Advanced Technology (ITMA), Universiti Putra Malaysia, 43400 UPM, Serdang, Selangor, Malaysia

**Keywords:** Nanohybrid, 3, 4-Dichlorophenoxyacetic acid, Layered double hydroxide, Anionic clay

## Abstract

A new layered organic–inorganic nanohybrid material, zinc-aluminum-3,4-dicholorophenoxyacetate (N3,4-D) in which an agrochemical, 3,4-dichlorophenoxyacetic acid (3,4-D), is intercalated into zinc-aluminum-layered double hydroxide (ZAL), was synthesized by coprecipitation method. A well-ordered nanomaterial was formed with a percentage loading of 53.5% (*w*/*w*). Due to the inclusion of 3,4-D, basal spacing expanded from 8.9 Å in ZAL to 18.7 Å in N3,4-D. The Fourier transform infrared study shows that the absorption bands of the resulting nanohybrid composed of both the 3,4-D and ZAL further confirmed the intercalation episode. Thermal analysis shows that ZAL host enhances the thermal stability of 3,4-D. Controlled-release experiment shows that the release of 3,4-D in the aqueous media is in the order of phosphate > carbonate > sulfate > chloride. These studies demonstrate the successful intercalation of the 3,4-D and its controlled release property in various aqueous media.

## Background

In modern agriculture, various agrochemicals such as pesticides, herbicides, and plant regulators are widely used for effective pest management and ensuring optimum crop yield. Most herbicide formulations deliver the bulk of the active agents in an immediately available form that can be readily released to the environment [1]. For highly soluble pesticides, these formulations may result in great pesticide losses shortly after application before the molecules have time to diffuse into soil aggregates and reach adsorption sites in soil colloids [2]. This phenomenon leads to pesticide residues in the food chain, and this, in turn, has adverse effects in humans including carcinogenic, mutagenic, and teratogenic effects [3].

Contamination of pesticides through volatilization, leaching, runoff, and the persistence of agrochemicals in aqueous media has become a concerning environmental issue [4,5]. In addition, agrochemicals are highly toxic to wildlife (especially mammals) and other organisms and can remain in the aquatic environment for a long time [6]. Much effort was done focusing on ways to reduce the usage of excessive agrochemicals by the development of less hazardous formulations, such as controlled release formulations, in which only a part of the active ingredient is in an immediately available form and the bulk of the herbicide is sorbed in an inert support [1,7]. This strategy is advantageous since it allows the gradual release of agrochemicals over time, besides preventing instant loss of agrochemicals through volatilization, leaching, and runoff [8]. Moreover, it requires less energy and manpower than the conventional methods, leading to decreased nontarget effect and increased safety for agrochemical applicators [9,10].

Clay has become one of the popular materials as a host of herbicides due to its unique properties such as high specific surface areas associated with their small particle size and ubiquitous occurrence in most soil and sediment environment [11-17]. One of the classes in the clay family is layered double hydroxide (LDH) or the so-called hydrotalcite-like compounds (HTs). This special material can be used as support in controlled-release formulations and has been proposed as the ideal solution to environmental problems caused by agrochemicals. LDHs or HTs are brucite-like layered materials with the general formula [M^II^_1 − *x*_ M^III^_*X*_(OH)_2_]^*x*+^(A^m−^)_*x*/*n*_·mH_2_O, where M^II^ and M^III^ are divalent and trivalent cations, respectively, and X^*n*−^ is the interlayer anion, which balances the positive charge generated by the presence of M^III^ in the layers. The layer charge is determined by the molar ratio *x* = M^III^/(M^III^ + M^II^) which can vary between 0.2 and 0.4 [18]. LDHs have attracted the attention of the industry and academia because of their anion-exchange capability [19], low cost, ease of preparation, environmental compatibility (especially in agricultural application), and potential use in pharmaceuticals, detergents, and food additives [20].

**Figure 1:**
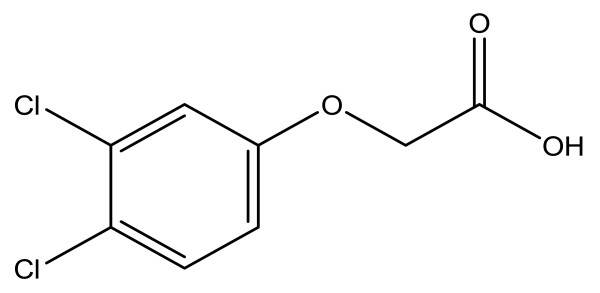
Molecular structure of 3,4-dichlorophenoxy acetic acid.

3,4-Dichlorophenoxy acetic acid (3,4-D) (Figure 1) is an organic anion used widely in modern agriculture to control weeds in paddy field and wheat and corn plantations [21].

In this study, the intercalation of 3,4-dichlorophenoxyacetic acid into the interlamellae of zinc-aluminum-layered double hydroxide (ZAL) was accomplished by a simple direct self-assembly method for the formation of a new organic–inorganic nanohybrid material. The physicochemical properties and the controlled release of the agrochemical were investigated and discussed.

## Methods

All chemicals used in this synthesis were obtained from various chemical suppliers and used without further purification. Zinc nitrate (Zn(NO_3_)_2_·6H_2_O, 98%, ChemPurPiekary Slaskie, Poland) and aluminum nitrate (Al(NO_3_)_3_·9H_2_O, 98%, ChemPurPiekary Slaskie, Poland) were used as the sources of cations while 3,4-dichlorophenoxy acetic acid (C_9_H_9_ClO_3_, 95%, Sigma-Aldrich Corporation, St. Louis, MO, USA) was used as the starting material of the guest anion. All solutions were prepared using deionized water.

### Synthesis of materials

The synthesis of Zn-Al-3,4D nanocomposites was performed by self-assembly method from a mixed aqueous solution of 0.1 M Zn(NO_3_)_2_·6H_2_O and 0.025 M Al(N0_3_)_3_·9H_2_O at various concentrations of 3,4D ranging from 0.0035 to 0.5 M. NaOH (2 M) was then added to the mixture with vigorous stirring under nitrogen atmosphere at a constant pH of 7.5 ± 0.02. The precipitate was aged for 18 h in an oil bath shaker at 70°C, filtered, thoroughly washed, and dried in a vacuum oven at 70°C. The resulting nanocomposite was finely ground, kept in a sample bottle, and stored in a vacuum desiccator for further use and characterization. A similar procedure was performed for the preparation of ZAL except the addition of 3,4D.

### Characterization

Powder X-ray diffraction (PXRD) patterns were recorded on a Rigaku model Ultima IV powder λ diffractometer (Rigaku Corporation, Tokyo, Japan) using filtered Cu-Kα radiation (*λ* = 1.540562 Å) at 40 kV, 20 mA, and 2° min^−1^. Fourier transform infrared (FTIR) spectra were recorded using a PerkinElmer ×1,725 spectrophotometer (PerkinElmer, Waltham, MA, USA) in the range of 400 to 4,000 cm^−1^. Finely ground 1% samples in KBr powder were compressed to obtain a pellet, and the pellet was then used to obtain the IR spectra. Thermogravimetric and differential thermogravimetric analyses (TGA/DTG) were carried out using a Mettler Toledo TGA/SDTA851 thermogravimetric analyzer (Mettler Toledo Inc., Columbus, OH, USA) with a heating rate of 10°C min^−1^ between 35°C and 1,000°C, under a nitrogen flow rate of 50 ml min^−1^. The elemental analysis was performed using a CHNS analyzer (model CHNS-932, LECO Corporation, St. Joseph, MI, USA) together with inductively coupled plasma atomic emission spectrometry using a PerkinElmer spectrophotometer (model Optima 2000DV) under standard condition.

## Results and discussion

### Powder X-ray diffraction

**Figure 2:**
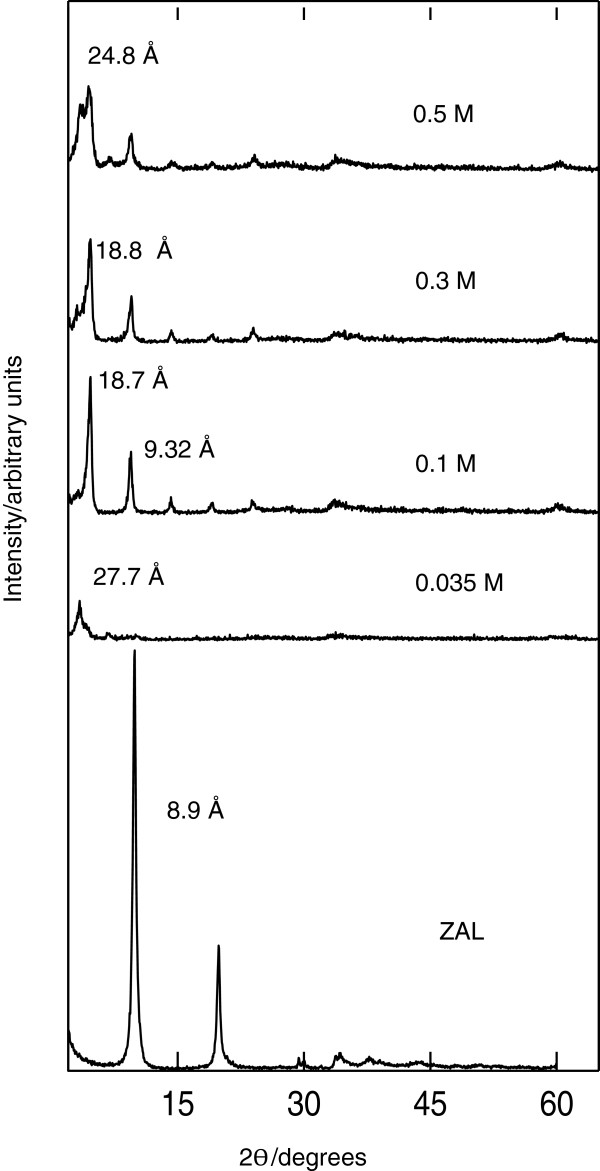
PXRD patterns of ZAL and its nanohybrids prepared at various concentrations of 3,4-D (0.035 to 0.5 M).

Figure 2 shows the PXRD patterns of the ZAL and its nanohybrid material, zinc-aluminum-3,4-dicholorophenoxyacetate (N3,4-D), prepared using various concentrations of 3,4-D from 0.0035 to 0.5 M. As shown in the figure, the basal spacing of ZAL, which contains nitrate ion as the counter anion in the interlayer, was recorded to be 8.9 Å which is in a good agreement with the sum of the thickness of the anion, NO_3_^−^ (4.1 Å), and the brucite-like layer (4.8 Å) [22]. The increasing basal spacing from 8.9 to 24.8 Å in the resulting nanocomposite, N3,4-D, was due to the inclusion of the new anion 3,4-D, which is bigger than nitrate, into the interlamellae space. This shows that 3,4-D has higher affinity toward ZAL compared to the counter anion (nitrate). When the concentration of 3,4-D was increased from 0.3 to 0.5 M, we observed that the reflection peaks at around 2*θ* = 0.4° became broad especially for 003 reflections showing a mix phase of the material due to the 3,4-D absorbed on the surface of ZAL. The best well-ordered nanocomposite was synthesized with 0.1 M which produced a sharp, symmetric, high-intensity peak, especially for 003 and 006 reflection peaks. This sample was then chosen for further characterization.

### FTIR spectroscopy

**Figure 3:**
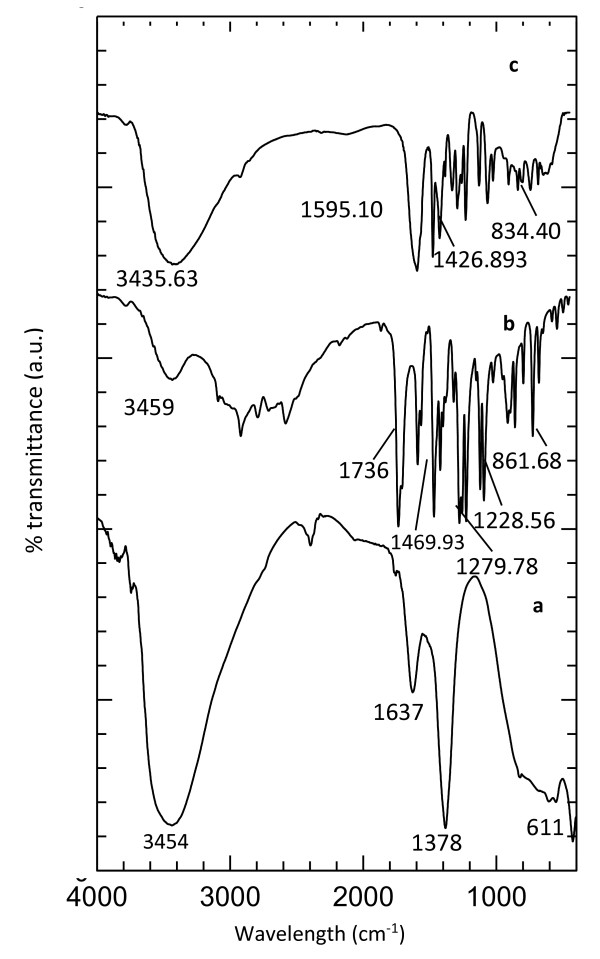
FTIR spectra of ZAL (a), pure 3,4-D (b), and N3,4-D nanocomposite (c).

The FTIR spectra for ZAL (Figure 3 (curve a)) showed a broad and strong band in the range of 3,200 to 3,600 cm^−1^ centered at 3,454 cm^−1^ which is due to the O-H stretching vibration of the inorganic layers and interlayer water molecules. Another common wave number for the LDH-like material is a band at 1,637 cm^−1^ which is assigned to the bending vibration of interlayer water molecules. For ZAL, a strong absorption centered at 1,378 cm^−1^ is assigned to the nitrate stretching vibration. A band in the lower wave number region corresponds to the lattice vibration mode such as the translation of Zn-OH at 611 cm^−1^ and the vibration of OH-Zn-Al-OH at 427 cm^−1^[23]. The FTIR spectrum of pure 3,4-D shows a broad band at 3,459 cm^−1^, which is attributed to the O-H stretching vibration. A band at 1,713 cm^−1^ is due to the C=O stretching. Bands at 1,469 and 1,400 cm^−1^ are attributed to the stretching vibration of aromatic ring C=C. Bands at 1,288 and 1,219 cm^−1^ are due to the symmetric and asymmetric stretching modes of C-O-C, respectively. A sharp band at 861 cm^−1^ is attributed to C-Cl stretching [24]. The FTIR spectra for the nanocomposite (N3,4-D) show a broad absorption band at around 3,400 cm^−1^ which arises from the stretching mode of OH groups in the brucite-like layer and/or physisorbed water. A band at 1,595 cm^−1^ is attributed to the carboxylate functional group of the intercalated 3,4 D anion. A band at 1,426 cm^−1^ can be attributed to the C=C bond vibration of the aromatic group. A band at 1,220 cm^−1^ corresponds to asymmetric and symmetric vibrations of C-O-C, respectively.

### Elemental analysis

Table 1 shows the elemental and organic content of ZAL and N3,4-D nanocomposites. As shown in Table 1, the molar ratio of Zn to Al in both ZAL and N3,4-D is 3.6 and 3.7, respectively, which is close to the initial value of the Zn to Al ratio in the mother liquor. The ZAL contains about 3.1% (*w*/*w*) nitrogen which is in agreement with the presence of a strong, sharp band at 1,378 cm^−1^ in the FTIR spectrum that corresponds to the nitrate group in ZAL. The percentage of 3,4-D intercalated into the interlayer of ZAL is 53.5% (*w*/*w*), estimated from the carbon content of about 23.2% (*w*/*w*), indicating that intercalation of 3,4-D has actually taken place.

**Table 1:** Basal spacing and chemical composition of Zn/Al-LDH (LDH) and its nanohybrid (N3,4-D)

**Sample**	***d *(Å)**	**Zn/Al ratio**	**Mole fraction (*x***_**Al**_**)**	***N *(%)**	***C *(%)**	**Anion**^**a **^**(% *w*/ *w *)**	**BET surface area (m**^**2 **^**g**^**−1**^**)**	**BJH desorption pore volume (cm**^**3 **^**g**^**−1**^**)**	**BET average pore diameter (Å)**
LDH	8.9	3.64	0.210	3.1	-	-	1.3	0.024	127
N3,4-D	18.7	3.70	0.233	-	23.24	53.5	3.0	1.240	66.67

^a^Estimated from CHNS analysis.

The surface area and porosity of ZAL and N3,4-D obtained by the nitrogen adsorption-desorption method are given in Table 1. The successful intercalation has increased the Brunauer-Emmett-Teller (BET) surface area from 1.3 m^2^ g^−1^ in ZAL to 3.0 m^2^ g^−1^ in N3,4-D. The change in pore texture with larger width, as a result of the modification by the intercalation of 3,4-D into the ZAL interlayer, which is in agreement with the expansion of basal spacing from the resulting nanohybrid (Figure 1) is thought to be the reason.

### Surface properties

**Figure 4:**
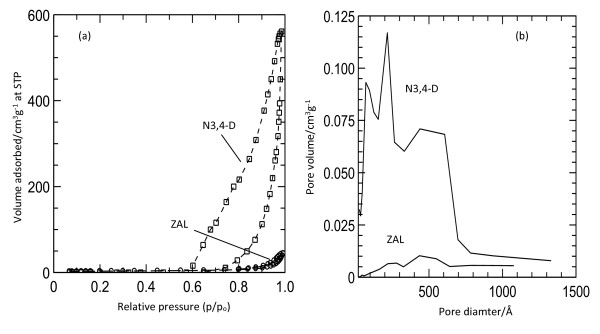
Nitrogen adsorption-desorption isotherms of ZAL and their nanohybrids (N3,4-D) (a) and pore size distribution (b).

The nitrogen adsorption-desorption isotherms (Figure 4) for ZAL and N3,4-D show Type IV material in the IUPAC classification, indicating a mesopore type of material. The adsorption branch of the hysteresis loop for the N3,4-D is wider than the one for LDH, indicating a different pore texture. This can be related to the expansion of basal spacing when nitrate is replaced by 3,4-D during the formation of the nanocomposite.

**Figure 5:**
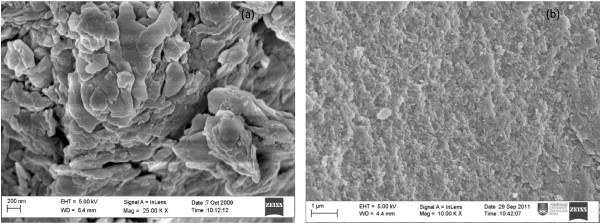
Surface morphology of (a) ZAL and N3,4-D (b).

Figure 4b shows the Barret-Joyner-Halenda (BJH) desorption pore size distribution for 3,4-D and its nanohybrid (N3,4-D). The summary of pore volume and pore diameter is given in Table 1. A sharp peak at 200.5 Å and a low-intensity sharp peak at 600.9 Å can be observed. On the other hand, LDH also showed a sharp peak at around 400 Å, and the pore size of LDH is lower compared to that of N3,4-D (Table 1). This may have resulted from the formation of interstitial pores between the crystallite, different particle sizes, morphology, and aggregation during the formation of the nanohybrid. The surface morphology of N3,4-D (Figure 5b) shows an agglomerate, porous, granular structure of N3,4-D compared to the nonporous morphology of ZAL (Figure 5a).

### Thermal analysis

**Figure 6:**
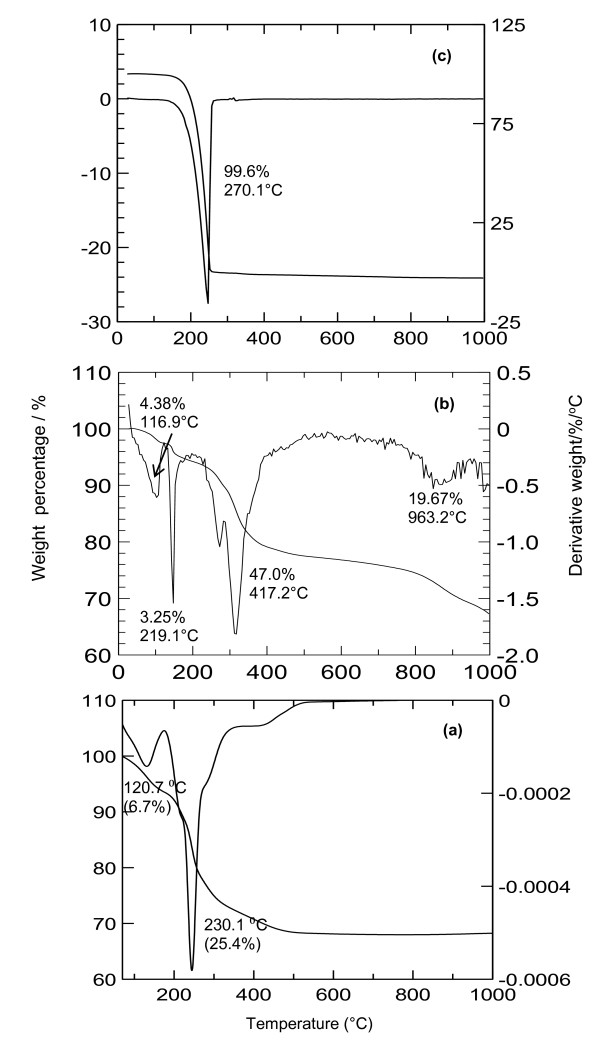
TGA-DTA thermograms of ZAL (a), pure 3,4-D (b), and N3,4-D nanocomposite (c).

The TGA-DTG profiles of ZAL, pure 3,4-D, and N3,4-D nanocomposites are shown in Figure 6. The TGA-DTG curves of N3,4-D reveal four weight losses occurring at 116.9°C, 219.1°C, 417.2°C, and 963.2°C that amounted 4.38%, 3.25%, 47.0%, and 19.7%, respectively. The first weight loss is due to the removal of surface-physisorbed water molecules, and the second stage is attributed to the removal of the interlayer anion and dehydroxylation of the hydroxyl layer. The third weight loss at 417.2°C corresponds to the major decomposition of the organic moiety in the interlayer of the nanohybrid, leaving only a relatively less volatile metal oxide. The weight loss of 6.7% that occurred at around 963.2°C is due to the decomposition of the more stable compound of the inorganic layered composition of the nanohybrid by combustion reaction [25]. The decomposition temperature for pure 3,4-D is 270.1°C, but the thermal stability of 3,4-D is greatly improved after intercalation between the LDH layer which is 417.2°C, implying that ZAL can be used as an alternative inorganic matrix for storing an active organic moiety with better thermal stability.

### Release profile of the 3,4-D into various aqueous solutions

**Figure 7:**
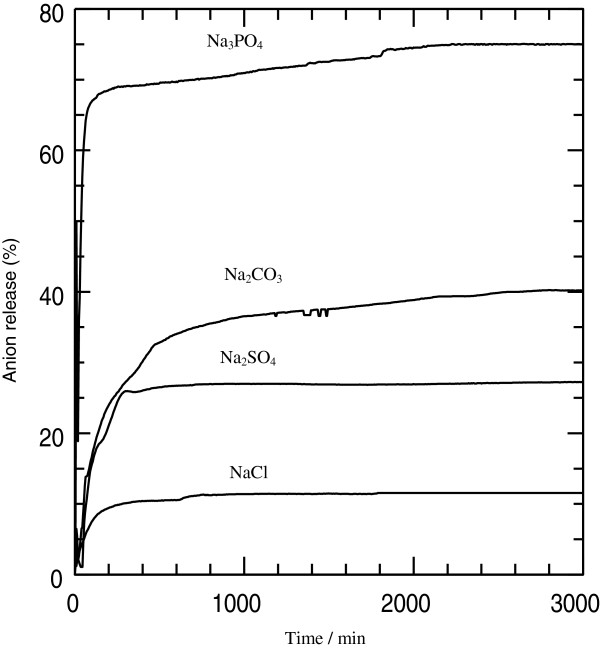
**Release profiles of 3,4-D from N3,4-D into 0.005 M aqueous solutions containing PO**_**4**_^**3−**^**, CO**_**3**_^**2−**^**, SO**_**4**_^**2−**^**, and Cl**^**−**^**.**

Release profiles of 3,4-D from the nanohybrid composite, N3,4-D, into various aqueous solutions, sodium phosphate, sodium carbonate, sodium sulfate, and sodium chloride (0.005 M), are shown in Figure 7.

The accumulated release of 3,4-D into various aqueous solutions containing phosphate, carbonate, sulfate, and chloride anions increased with contact time. The release of the 3,4-D from the nanohybrid was fast for the first 200 min, followed by a slower one subsequently before reaching the saturated release at approximately 300 and 500 min for PO_4_^3−^ and Cl^−^ and CO_3_^2−^ and SO_4_^2−^, respectively.

Saturated release of the anions is in the order of phosphate > carbonate > sulfate > chloride with percentages of saturated release of 75%, 40%, 27%, and 11%, respectively. The highest saturated release of 3,4-D in the PO_4_^3−^ aqueous solution is due to the high charge density of the anion (PO_4_^3−^), whereas the lowest saturated release of 3,4-D was in the aqueous solution containing Cl^−^. This shows that the saturated release for the aqueous media toward the anion encapsulates in LDH agreed with the previous work by Miyata et al. [26]. This result suggests that the charge density of the anion to be exchanged with 3,4-D plays a vital role in determining the saturated release of the 3,4-D from the nanohybrid into the aqueous media.

### Kinetic release

For quantitative analysis, the data from the release study were fitted into zeroth-order (Equation 1), first-order (Equation 2), parabolic diffusion (Equation 3), and pseudo-second-order kinetic models (Equation 4). The equations are given as follows:

(1) $$C_{t}=\mathrm{kt}+c$$

(2) $$-\log\left(1-C_{t}\right)=\mathrm{kt}+c$$

(3) $$C_{t}/C_{\mathrm{eq}}=c+kt^{0.5}$$

(4) $$t/C_{t}=1/k_{2}C^{2}{}_{\mathrm{eq}}+\left(1/q_{e}\right)t$$

**Figure 8:**
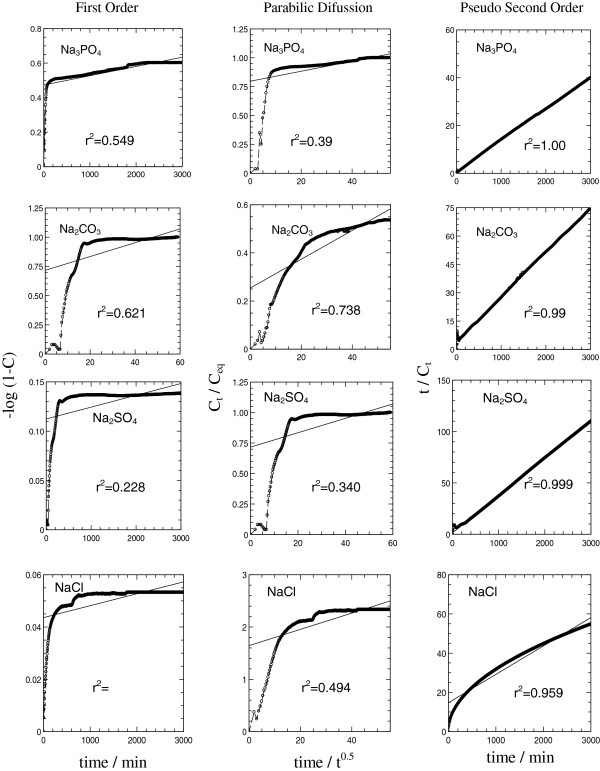
**Release profiles of 3,4-D.** Fitting the release data of 3,4-D from the nanohybrid into various aqueous media (Na_3_PO_4_, Na_2_CO_3_, Na_2_SO_4_, and NaCl (0.005 M)) using first-order, parabolic diffusion, and pseudo-second-order kinetic models.

Figure 8 shows the release profiles of 3,4-D fitted to the first-order, parabolic diffusion, and pseudo-second-order kinetic models. The corresponding rate constant, *k*, together with regression value, *r*^2^, obtained from the fittings is summarized in Table 2. The release of 3,4-D into SO_4_^2−^, CO_3_^2−^, PO_4_^3−^, and Cl^−^ aqueous solutions was formed to follow the pseudo-second-order kinetic models with *r*^2^ close to 1. The *t*_½_ values, the time it takes for the concentration of 3,4-D to be at half of the accumulated saturated release, were found to be 39, 56, 74, and 78 min for 3,4-D release in phosphate, carbonate, sulfate, and chloride aqueous solutions, respectively. The *t*_½_ values are in the order of phosphate < carbonate < sulfate < chloride which followed the release rate of the organic moieties in the aqueous solution mentioned above, as *t*_½_ is inversely proportional to the release rate [27].

**Table 2:** **Rate constant, half time, and correlation coefficient (*r***^**2**^**) value**

**Aqueous solution (0.005 M)**	**Zeroth-order *r***^**2**^	**First-order *r***^**2**^	**Parabolic diffusion**			**Pseudo-second-order**		
	**(3,000 min)**	**(3,000 min)**	***r***^**2**^	***k *(×10**^**−3**^**)**	***c***	***r***^**2**^	***t***_**1/2 **_**(min) *k *(×10**^**−4**^**)**	***c***
Na_3_PO_4_	0.315	0.549	0.390	15.50	0.797	1.000	39 2.458	0.698
Na_2_CO_3_	0.567	0.621	0.738	5.99	0.254	0.999	66 2.424	0.391
Na_2_SO_4_	0.215	0.228	0.340	4.32	0.717	0.999	74 2.235	1.360
NaCl	0.322	0.336	0.494	5.90	1.640	0.959	78 2.146	1.470

Obtained from the fitting of the data from 0 to 3,000 min of 3,4-D in the LDH interlayer into the aqueous solution containing various anions, phosphate, carbonate, sulfate, and chloride, by first-order, parabolic diffusion, and pseudo-second-order kinetics models.

## Conclusions

A herbicide compound, 3,4-D, was successfully intercalated into the layer of ZAL for the formation of a new organic–inorganic hybrid nanocomposite, N3,4-D, which shows a potential to be used as a controlled-release formulation in agrochemicals. The interlayer spacing of LDH increased from 8.9 to 18.72 Å in the N3,4-D due to the inclusion of 3,4-D into the Zn-Al-LDH interlayer space. Release of 3,4-D from the Zn-Al-layered inorganic host follows pseudo-second-order kinetic models with regression values of 0.959 to 1. This study suggests the possibility of zinc-aluminum-layered double hydroxide to be used as a carrier host for 3,4-D for the generation of environmentally friendly agrochemicals.

## Competing interests

The authors declare that they have no competing interests.

## Authors’ contributions

SAISMG wrote the paper, performed the experiments, and analyzed the data. MZH and SHS conceived the study, participated in the design and coordination of the scientific team, and assisted in drafting the manuscript. All authors read and approved the final manuscript.
